# Supplementary figures and images for: Ethnomedicinal and Ethnobotanical Survey in the Aosta Valley Side of the Gran Paradiso National Park (Western Alps, Italy)
Source: Plants (Basel). 2022 Jan 9;11(2):170. doi: 10.3390/plants11020170 (PMC8778718; doi:10.3390/plants11020170)

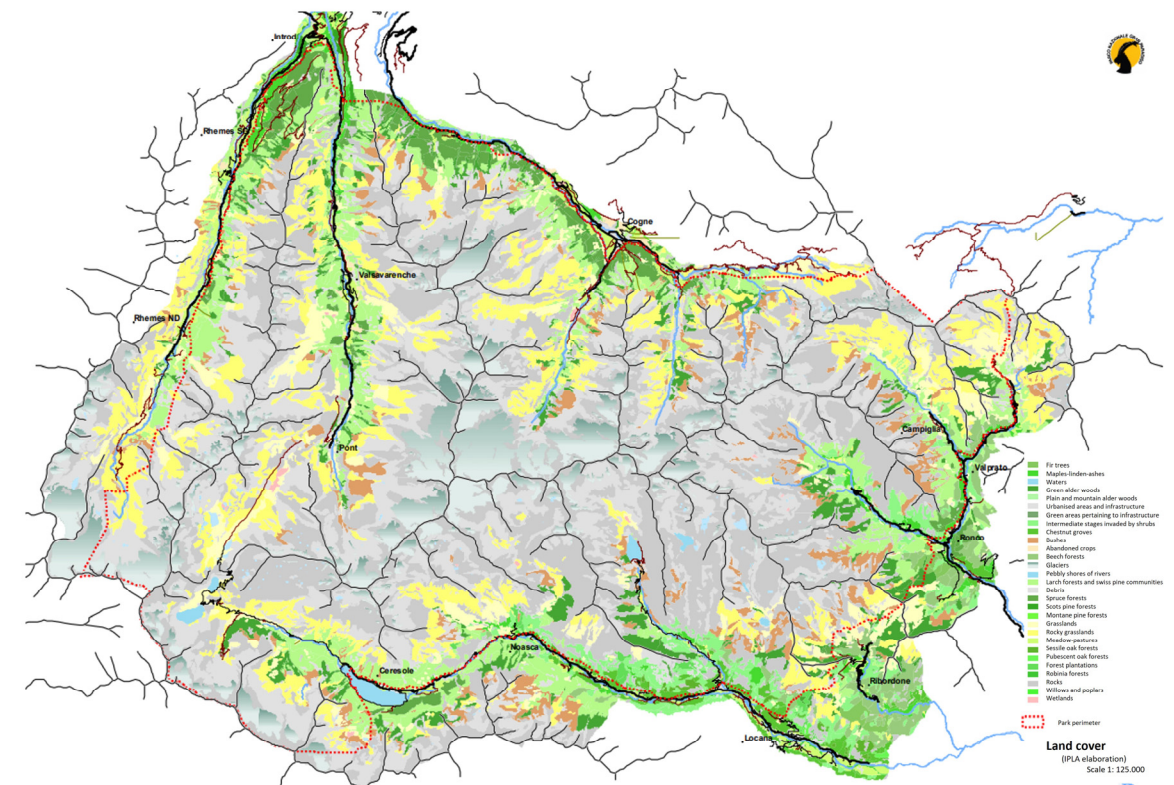

**Figure S1.** Land use and vegetation map of the Gran Paradiso National Park.

Supplement: Supplementary file 1 [file plants-11-00170-s001.zip › plants-1524754-supplementary.pdf]
